# Cognitive Effects of Mindfulness Training: Results of a Pilot Study Based on a Theory Driven Approach

**DOI:** 10.3389/fpsyg.2016.01037

**Published:** 2016-07-12

**Authors:** Lena Wimmer, Silja Bellingrath, Lisa von Stockhausen

**Affiliations:** Department of Psychology, University of Duisburg-EssenEssen, Germany

**Keywords:** mindfulness, attention, cognition, meditation, cognitive flexibility, cognitive inhibition, children, school

## Abstract

The present paper reports a pilot study which tested cognitive effects of mindfulness practice in a theory-driven approach. Thirty-four fifth graders received either a mindfulness training which was based on the mindfulness-based stress reduction approach (experimental group), a concentration training (active control group), or no treatment (passive control group). Based on the operational definition of mindfulness by Bishop et al. (2004), effects on sustained attention, cognitive flexibility, cognitive inhibition, and data-driven as opposed to schema-based information processing were predicted. These abilities were assessed in a pre-post design by means of a vigilance test, a reversible figures test, the Wisconsin Card Sorting Test, a Stroop test, a visual search task, and a recognition task of prototypical faces. Results suggest that the mindfulness training specifically improved cognitive inhibition and data-driven information processing.

## Introduction

Effects of mindfulness have inspired increasing research activities over the last two decades. This research has documented beneficial effects of mindfulness-based interventions on well-being and mental as well as physical health in adult populations (Brown and Ryan, 2003). Following Bishop et al. (2004) and Kabat-Zinn (2005), we understand mindfulness as a non-judgmental, accepting awareness of moment-by-moment experience. Formal meditation practices are at the core of mindfulness-based interventions, among them the most popular intervention, Mindfulness-Based Stress Reduction (MBSR, Kabat-Zinn, 2005). Despite the fact that mindfulness is considered a special form of attention, i.e., a cognitive phenomenon in itself, research into the cognitive effects of mindfulness falls far behind the extent of pertinent clinical research (Chiesa et al., 2011). Furthermore, studies examining the impact of mindfulness on cognitive functions in the developing brain have been especially scarce, even though the introduction of mindfulness-based interventions in school settings has increased rapidly over the last years (Meiklejohn et al., 2012). Therefore, the present study aimed to investigate cognitive effects of a mindfulness intervention in fifth graders^1^. The adolescent brain is characterized by profound maturational changes especially in the prefrontal cortex (PFC), which is responsible for higher cognitive functions as well as the cognitive control of emotions and motivation (Paus et al., 2008). This process starts in puberty (between 10 and 12 years of age) and continues into the early twenties. Riggs et al. (2014) therefore suggest that the impact of prevention strategies focusing on the promotion of behavioral and cognitive control could be increased, if the time course of PFC development is considered. Supporting this notion, a recent meta-analysis on the effects of mindfulness-based interventions for children and adolescents in school settings (Zenner et al., 2014) found an overall effect size of Hedge's *g* = 0.40 across all studies and domains included (cognitive performance, emotional problems, stress and coping, resilience, third person ratings), but an effect size of *g* = 0.80 for cognitive performance. The present study is based on an operational definition of mindfulness provided by Bishop et al. (2004), which links mindfulness to traditional concepts of cognitive psychology and well-established cognitive tasks. Before describing the conceptualization in detail, we will first provide an overview of research on the cognitive effects of mindfulness training. Following established classifications of cognitive abilities (e.g., Anderson, 2010; Chiesa et al., 2011), the subsequent section outlines empirical studies that investigated the impact of mindfulness on attention, working memory, cognitive inhibition, and creative problem solving. These competencies are discussed in a quasi-hierarchical order in that fundamental processes, such as attention, provide the precondition for higher-order processes, such as creative problem solving.

### Overview of existing studies

A range of investigations drew on the tripartite model of attention by Posner and Petersen (Posner and Petersen, 1990; Petersen and Posner, 2012) in order to test effects of mindfulness meditation on attention. Attention, as conceptualized by Posner et al., consists of the *alerting network*, the *orienting network*, and the *executive attention network*. The Attention Network Test (ANT; Fan et al., 2002) was developed to empirically test the performance of the three subsystems. Studies investigating the relationship between mindfulness and performance in the ANT yielded a complex pattern of results: Different studies showed improvement in the executive attention network after mindfulness meditation training (e.g., Tang et al., 2007; van den Hurk et al., 2010; Ainsworth et al., 2013; Elliott et al., 2014). van den Hurk et al. (2010) also found that expert meditators were superior to control participants in the orienting network. Participants of Jha et al. (2007) showed an ameliorated performance in the orienting network after an 8-week MBSR course and in the alerting network after a more intense 1-month meditation retreat. Empirical evidence (Elliott et al., 2014) suggests that beneficial effects of mindfulness meditation on attention go back to a decoupling of the alerting and executive attention networks.

Numerous other studies investigated effects of mindfulness meditation on attention without directly relating to Posner and Petersen's model (Posner and Petersen, 1990; Petersen and Posner, 2012). Sustained attention, measured with various computer-based tests such as the Internal Switching Task^2^ or the Vigil Continuous Performance Test (The Psychological Corporation, as cited in Anderson et al., 2007) improved after intensive meditation trainings (e.g., at least 10 days of intensive retreat covering 10 h of daily meditation practice, Chambers et al., 2008; Lutz et al., 2009; MacLean et al., 2010). In contrast, sustained attention did not improve after a less intensive MBSR course (Anderson et al., 2007).

Results regarding the impact of mindfulness practice on selective/orientational attention or attention switching are less clear-cut. In a selective attention task (Posner, 1980), meditators were less distracted by invalid cues than non-meditators (Hodgins and Adair, 2010). However, other studies with cross-sectional (Chan and Woollacott, 2007) as well as experimental designs (Anderson et al., 2007; Chambers et al., 2008; see above) suggest that orientational attention or attention switching is not enhanced by mindfulness practice.

The impact of mindfulness meditation on working memory, on the other hand, was investigated with converging results. Two studies measured participant's working memory capacity by means of the digit span backward and forward of the Wechsler Adult Intelligence Scale before and after completing a mindfulness retreat (Chambers et al., 2008; see above) or a less intensive training of focused attention meditation (Zeidan et al., 2010). Further studies used the operation span task (Unsworth et al., 2005) as an indicator of working memory capacity. These studies report at least a tendency for working memory capacity to improve more after Mindfulness-Based Mind Fitness Training (Jha et al., 2010) or a 2-week mindfulness training^3^ (Mrazek et al., 2013) than in control conditions. The degree of experience in mindfulness practice and of training intensity seems to be conducive to beneficial effects in this respect (Chiesa et al., 2011).

Several studies support the assumption that mindfulness practice fosters cognitive inhibition. In two cross-sectional studies, meditators outperformed control participants in Stroop performance (Chan and Woollacott, 2007; Moore and Malinowski, 2009; see also Malinowski, 2013). However, Anderson et al. (2007) could not confirm specific effects of an MBSR course on Stroop performance. Further evidence of mindfulness-based improvement of cognitive inhibition comes from an experimental study by Zanesco et al. (2013).

Regarding creative problem solving, existing studies support the notion that mindfulness may facilitate insight problem solving as opposed to non-insight problem solving (Ren et al., 2011; Ostafin and Kassman, 2012). Comparing two kinds of meditation exercises, open monitoring, and focused attention, both of which are part of mindfulness trainings (Lutz et al., 2008), Colzato et al. (2012) showed that open monitoring meditation enhanced divergent thinking, but that focused attention meditation did not promote convergent thinking. Enhanced divergent thinking through meditative practice was also demonstrated by Ding et al. (2014). Positive affect is assumed to mediate the impact of mindfulness meditation on creativity (Colzato et al., 2012; Capurso et al., 2014; Ding et al., 2014).

The following studies investigated mindfulness-based cognitive effects in children: Napoli et al. (2005) found specific benefits in the selective attention subtest of the Test of Everyday Attention for Children (Tea-Ch; Manly et al., 2001) after participants had received the Attention Academy Program^4^, but no benefits in the sustained attention subtest. Flook et al. (2010) investigated the impact of mindful awareness practices (MAPs) on executive functions, as assessed by teachers and parents using the Behavior Rating Inventory of Executive Function (BRIEF; Gioia et al., 2000). Overall, participants in the MAPs condition did not develop differently than controls from pre- to post-test, but those children in the MAPs condition with initially poor executive functions showed greater improvement at posttest compared to controls. In a study by Franco Justo (2009), participation in a meditation program significantly enhanced students' creativity as measured with the Torrance Test of Creative Thinking (TTCT; Torrance, 1974), whereas the control group did not improve.

Taken together, existing research supports the notion that mindfulness can enhance cognitive functioning on basic as well as higher-order levels. But up to now, pertinent evidence rests on an unstable foundation. Existing studies vary in their inclusion of control groups (active/passive), in their design (cross-sectional/training study), in participants' expertise (novices/experts), the length/intensity of trainings and in the operationalization of dependent variables. Furthermore, the results of Jensen et al. (2012) point to the possibility that some effects of MBSR on attention, especially on reaction-time-based measures, may be traced back to increased attentional effort rather than to increased attentional abilities *per se*. Moreover, a general shortcoming lies in a mostly data-driven approach that fails to consider theoretical models of mindfulness in deriving hypotheses and interpreting results. The lack of a theoretical basis also impedes the integration of findings across studies. Finally, the cognitive effects reported above were mainly studied in adults rather than children. Similar to the studies conducted with adults, the generally promising evidence regarding children and adolescents is limited by several methodological shortcomings, as acknowledged in different reviews of the empirical research in this field (cf. Burke, 2010; Greenberg and Harris, 2012; Harnett and Dawe, 2012; Zenner et al., 2014), among them the diversity of study samples, variation in implementation and exercises, negligence of objective outcome measures, and lack of active control groups.

### Approach and hypotheses of the present study

The present study aims to address some of the shortcomings mentioned by implementing a theory-driven approach, by investigating objective outcome measures and including an active and a passive control group. Our approach is based on Bishop et al.'s (2004) two-component model of mindfulness. The first component is the self-regulation of attention, so that attention remains focused on the immediate present experience. The second component, orientation toward experience, can be described as an attitude of curiosity for, openness to, and acceptance of the present moment. We use the first component to derive hypotheses on cognitive effects of mindfulness practice. According to Bishop et al. (2004) self-regulation of attention promotes specific fundamental cognitive processes: (a) sustained attention, that is, the ability to attend to specific external or internal stimuli for an extended period of time without being distracted, (b) attention switching or cognitive flexibility, that is, the ability to deliberately change the focus of attention in response to a change in demands, (c) inhibition of secondary elaborative processing or cognitive inhibition, that is, the ability to suppress automatic responses if they interfere with current demands, and (d) data-driven information processing or the “beginner's mind” as opposed to schema-based information processing.

In describing sitting meditation as a core component of mindfulness practice, we will now explicate how practicing mindfulness is assumed to facilitate the aforementioned cognitive processes. In addition we will describe how the proposed processes can be assessed with cognitive tasks. In sitting meditation, attention is continuously focused on one's own breath while maintaining a calm, upright position. Meditators continuously observe their incoming and outflowing breath without interfering. The practice of maintaining awareness of the sensations connected with the breath is assumed to require as well as to foster *sustained attention* (Bishop et al., 2004) while involving the alerting network (Malinowski, 2013). Sustained attention can be measured with vigilance tests, which consist of very simple but time-sensitive tasks that have to be executed for an extended period of time. As soon as the mind strays from the breath, the default mode network takes over. Due to attention monitoring with the help of the salience network, the source of distraction is supposed to be noted. The executive network is crucial in disengaging from the distracting stimuli. Finally, attention is directed back to the object of interest, while the orienting and the executive network enable attention shifting (Malinowski, 2013). The practice of continuously orienting attention back to the breath is assumed to promote attention switching or *cognitive flexibility*, which can be assessed, for example, with the perseverative component of the Wisconsin Card Sorting task (Berg, 1948)^5^. This type of task induces a repeated pattern of similar responses and then changes demands in such a way that the earlier responses fail and have to be abandoned and changed. The process of immediately redirecting attention requires that the distracting stimuli, for instance, emerging thoughts, are considered mere (mental) events that are noticed but not reflexively acted upon. This means that impulses of automatic responding are inhibited. Consequently, breathing meditation is assumed to support *cognitive inhibition*. This ability can be assessed, for example, with a Stroop test, where automatic lexical access to the meaning of a color word must be inhibited in order to specify the color of the ink or print. The Stroop test has been used in several investigations of mindfulness (see above: e.g., Anderson et al., 2007; Chan and Woollacott, 2007; Moore and Malinowski, 2009). Finally, refraining from irrelevant elaborations saves attentional resources, so that the actual state of affairs at the current moment can be fully experienced. In this way, mindfulness practice is assumed to facilitate what is called a “beginner's mind,” or, in cognitive terms, *data-driven rather than schema-based information processing*. This predicted effect of mindfulness practice can be assessed with tasks where data-driven processing has an advantage over the use of schemata, as in detecting objects in unexpected settings (e.g., Biederman et al., 1973). Using a comparable paradigm, Anderson et al. (2007) showed that an increase in mindfulness is associated with an enhanced ability to discover objects in unexpected contexts.

As elaborated in the model by Bishop et al. (2004) and as outlined above, mindfulness practice is expected to promote the aforementioned cognitive abilities. In order to test specific cognitive effects of mindfulness practice, the present study contrasted the mindfulness intervention with a concentration training in an active control group. This training was not based on insight into one's attentional processes and how to regulate them but rather trained concentration on a behavioral level (see below). It was expected that participants in this group might also improve cognitive competencies. Finally, mere effects of schooling and maturation which could also lead to improvements in the described cognitive skills were controlled for by including a passive control group that received no intervention. The passive control group was predicted to improve to a smaller degree since both interventions were assumed to be effective beyond mere schooling and maturation. Cognitive abilities were assessed in a pre-post design, immediately before and after the intervention period.

The mindfulness training was based on the Mindfulness-Based Stress Reduction approach by Kabat-Zinn (2005), which has been repeatedly shown to be effective in clinical settings (Baer, 2003; Grossman et al., 2004; Hofmann et al., 2010; Keng et al., 2011)^6^ and which provides a structured training intervention. Adaptations of this approach for children and school settings have been developed and described by Greenland (2010), Kaltwasser (2008), and Kuyken et al. (2013). All our participants attended the fifth grade of a Gymnasium^1^ and were novices to the training methods used. This age category was selected because at this age children develop self-reflexive and metacognitive abilities which are required for (and benefit from) mindfulness training (Jankowski and Holas, 2014). Therefore, this age seems to be the earliest stage in life where a training of mindfulness in the proper sense might be fruitful, although intervention programs for younger children exist as well (Semple et al., 2006; Goodman and Greenland, 2009; Snel, 2013).

Since the extent of mindfulness practice, i.e., the invested time, has been identified as one of the most important predictors of program effectiveness (Zenner et al., 2014), we opted for a fairly intensive and extensive exercise program which comprised mindfulness interventions twice a week over the duration of 4 months.

To sum up, we predicted improved sustained attention, cognitive flexibility, cognitive inhibition, and data-driven information processing after mindfulness training. If mindfulness training has specific cognitive effects, participants should outperform those who received a non-mindfulness concentration training (active control group). Both interventions were supposed to result in a better performance than mere schooling and maturation (passive control group).

## Materials and methods

The study was approved by the ethics committee of the University of Duisburg-Essen, Faculty of Engineering.

### Participants

Participants were recruited in a three-step procedure. First, two schools in the city of Essen, Germany, were sent an invitation letter informing about the general aims and methods of the study. The Gymnasium Essen Nord-Ost indicated that they were basically interested in participating. Second, we selected two parallel classes whose class teachers were willing to participate in the study. Third, parents were informed about the training and were asked for consent. In the end, 34 fifth graders (16 male and 18 female participants, mean age 10.80 years at the beginning of the training period, *SD* = 0.53) from the Gymnasium Essen Nord-Ost, Essen, Germany, volunteered for the study. Sixteen participants were randomly assigned to the mindfulness intervention group (divided into two groups of eight students) and eight participants were assigned to a concentration training group. Randomization was implemented by having each child draw a lot that assigned them to one of the experimental conditions (mindfulness vs. concentration training). The experimental group consisted of eight boys and eight girls, in the active control group there was one boy and seven girls. The passive control group with no intervention consisted of 10 participants from a parallel class of the same school. In exchange for their participation these children received a book voucher worth €25 after finishing the second series of cognitive tests. The passive control group consisted of seven boys and three girls. All parents and students gave their informed consent.

### Interventions

The training was led by the first and the last author of this article. A team of five tutors plus the first and the last author ran the interventions. Three of the tutors were teacher trainees; two possessed a BA or MA in a pedagogic discipline. None of them had previous experience with mindfulness practice. The five tutors received extensive training with regard to the theoretical concept, self-practice and teaching of mindfulness. Fidelity of intervention delivery was secured by the regular presence of either the first or the last author in the training sessions and by weekly team supervisions where written protocols of the previous week's sessions, possible problems and the detailed program of the upcoming sessions were discussed. Each individual intervention session was led by at least two instructors. In order to avoid instructor effects, the composition of teams changed after three sessions and instructors rotated across intervention groups.

#### Mindfulness training

The mindfulness training was based on the well-established MBSR method (Kabat-Zinn, 2005). We also drew on an adapted version for children by Greenland (2010). The intervention comprised two essential exercises, sitting meditation, and the bodyscan. In sitting meditation, the aim is to constantly focus on one's own breath while letting go of arising thoughts or emotions. The training started with practicing times of 3 min. Later on, its duration was extended to 10 min. During the bodyscan, learners slowly guide their attention through the whole body, from the toes to the top of the skull. As it became apparent that the children were overtaxed with a complete scan, the instruction was split into an upper and a lower body part and these two were practiced in turns. Duration of these partial bodyscans varied between 5 and 15 min.The two exercises were assumed to promote sustained attention, cognitive flexibility, cognitive inhibition, and data-driven processing as outlined above. Further exercises aimed at raising awareness of the relations between sensations, their evaluation, concurrent or resulting emotions, and behavior. For instance, during the *melting ice exercise*, participants were holding an icecube in their palms for as long as possible. Meanwhile, they observed the interplay between sensations, emotions, thoughts, and behavioral tendencies without reflexively reacting to them. In the *What's behind my back-exercise*, a child received an object into their hands, which were held behind their back. The child was to describe the object in an unbiased, data-driven manner to the rest of particpants (e.g., the surface feels round, cold and smooth rather than the object feels like a watch), so that the others could guess the object. This exercise was hypothesized to foster data-driven information processing. Each individual session was started off with one or two of the yoga exercises proposed by Kabat-Zinn (2005). Sitting meditation or a bodyscan were practiced in each session, all other practices occurred only once or twice during the whole training. The training took place twice a week, once for 60 min, and once for 90 min, so that the children received roughly 150 min of treatment each week. The children were not asked to practice at home, in order to ensure standardization; hence, every child received the same amount of training. Trainings were held as part of the regular class time. One of the weekly sessions took place instead of regular remedial teaching, the other one replaced an elective course.

#### Control groups

The design of the study included an active and a passive control group. In order to identify cognitive effects that were specific to mindfulness training, the active control group received a training of cognitive ability, namely the German Marburg Concentration Training (Krowatschek et al., 2007, 2011), which is widely used in the German-speaking area to improve concentration skills in school children. Dreisörner (2004) as well as Hahnefeld and Heuschen (2009) report reductions in hyperactivity and attention deficits as a consequence of the training. It uses verbal self-instruction and principles from cognitive behavior therapy in order to promote self-regulation, autonomy, systematic problem solving, and rational error treatment while reducing impulsive behavior. Learning strategies, text comprehension and memory are practiced partly individually, partly in groups. Relaxation exercises based on autogenous training complement the Marburg Concentration Training. The intervention for this control group was based on the advanced exercises from the children's version (Krowatschek et al., 2011) and on the easier exercises from the adolescents' version (Krowatschek et al., 2007), since the age of our participants was just at the intersection of the target groups of both versions. The concentration training took place in parallel time slots to the mindfulness training, i.e., twice a week, once for 60 min, and once for 90 min, replacing regular remedial teaching and an elective course.

In order to control for effects of maturation and schooling the passive control group did not receive any experimental treatment.

### Materials

Each of the cognitive skills outlined above was measured with at least one computer-based test. The programming software used was ExperimentBuilder (SR Research Ltd., 2011). All tests were sucessfully pretested with a completely different sample of six children (whose ages ranged from 9 to 13 years). The pretest confirmed that all tests were easy to comprehend and were therefore appropriate for this age. Furthermore, manuals of the WCST-64 (Kongs et al., 2000) and that of an established version of the Stroop test which closely resembles the version used here, namely the color-word inference test by Bäumler (1985), provide normative data for children at the age of our target group.

#### Sustained attention

We measured sustained attention using a vigilance test which resembled the Moving Bar task, a subtest of the German Test of Attentional Performance (Zimmermann and Fimm, 2009). Participants observed a bar oscillating between two positions. Most of the time, the distance between the positions was fairly small. Only in the infrequent case of a large distance were participants expected to press a button. Every bar appeared for 643 msec, with an interstimulus interval of 170 msec. The experimental block contained 702 bars, of which 18 required pressing the button. The whole procedure took about 20 min. Participants were instructed to respond as fast and as accurately as possible. The lower the number of errors and the faster the responses, the higher the score of sustained attention.

#### Cognitive flexibility

Two measures indicated cognitive flexibility. First, participants were presented with six reversible figures, which appeared, one after another, on a computer screen. The pictures featured at least two specific, mutually exclusive interpretations like the famous rabbit-duck illusion (for an illustration see Jastrow, 1899, p. 312). They were taken from printed books and webpages. Participants were presented with different pictures during pre- and post-test. While looking at each item, participants were asked to indicate what the picture represented. They were told to find as many alternatives as possible without making up any solutions. High cognitive flexibility was indicated by a high number of correct answers. According to Hodgins and Adair (2010), meditators identified more alternative perspectives of reversible images in a similar task than non-meditators.

Secondly, participants completed the WCST-64 (Kongs et al., 2000). In this test, participants sort 64 playing cards according to an initially unknown criterion such as color, form or number of geometric objects displayed. The correct criterion is to be discovered with the help of feedback given after each trial and subsequent systematic testing. After a sequence of 10 correct solutions the criterion changes without announcement. High cognitive flexibility is indicated by faster recognition of the criterion change and by a comparatively lower sum score of perseverative errors, i.e., errors caused by sticking to an incorrect sorting criterion.

#### Cognitive inhibition

The level of cognitive inhibition was assessed using a classic Stroop Color-Word Interference Test. Color words were displayed either in the color they designated or in a different color. Responses were collected with a response pad with colored keys. Participants were to press the key of the respective display color as quickly and as accurately as possible, irrespective of the meaning of the color word. After a practice block featuring eight items, participants passed through 36 experimental trials. In this test, cognitive inhibition manifests itself in comparatively faster and more correct responses to items lacking a compatibility of word meaning and color.

#### Data-driven information processing

Two tasks assessed the capacity for data-driven processing. Firstly, a recognition task was designed based on work by Solso and McCarthy (1981), who showed that prototypical faces tend to be regarded as familiar, even if they have never been seen before. This incorrect recognition of prototypical faces, normally displayed by healthy adults, can be regarded as schema-based information processing, because participants rely on a feeling of familiarity and a schema that links familiarity with previous exposure. They do not rely on the actual facial features of the stimulus. In the present study, we made use of the prototypicality of morphed faces, which are also studied in research on attractivity (for a review, see Halberstadt, 2006). During an acquisition phase, participants were presented with 10 photographs of adult male faces, which appeared, one after another, on a computer screen. Each photograph was displayed for 10 s. Pictures were taken from the AR face database (Martinez and Benavente, 1998; Martinez and Kak, 2001; Ding and Martinez, 2010). Only faces without glasses and beard were selected. Participants were instructed to accurately memorize each image. After the acquisition phase there was a 5-min break, during which the measure of cognitive inhibition, the Stroop Color-Word Interference Test, was sampled (as outlined above). Then followed the recognition phase of the facial recognition task. Again, 10 faces were shown, one after another, to the participants. Five of these faces were original faces that had been presented during acquisition, while the remaining five faces were new. Two of the new faces were faces from the AR database. The remaining three pictures were morphs of faces from the acquisition phase; they differed in the number of faces involved: One picture was a morph of two faces, one of four faces, and one was a morph of all of the ten faces presented during acquisition. Generally, a morph becomes more prototypical the more faces are involved (morphs were created with the software FantaMorph 5 deluxe; Abrosoft, 2012). Participants were asked to indicate as quickly and accurately as possible whether they had seen the faces during the acquisition phase. A high level of data-driven information processing was reflected by relatively faster and more correct rejections of morphed faces, since these rejections are indicative of the ability to suspend the automatic tendency to falsely recognize prototypical faces.

The second measure of data-driven information processing was based on a visual search paradigm by Biederman and colleagues (Biederman et al., 1973, 1982). Participants first saw an object (prime) for 5 s, followed by a picture of an everyday scene taken from a children's picture book (e.g., a platform at a station, a pond with ducks). They were asked to indicate as quickly and as accurately as possible whether the target picture included the prime or not. The target picture either contained the prime in its original (and expectable) position such as a cloud in the sky (25% of all trials), or it contained the object in a totally unexpected position such as a cloud in front of a wall (25% of all trials), or it did not contain the prime object at all (50% of all trials). A practice block of four trials preceded the experimental block with 32 trials (i.e., eight trials with the prime object in expected position, eight in unexpected position, and 16 blank trials which did not show the prime object). In those trials where primes appeared in an unexpected position, they were evenly distributed over the four quadrants of the screen in order to avoid stereotyped responses. Data-driven information processing was reflected by comparatively better performance in detecting objects even in unexpected places and in correctly rejecting blank trials.

### Procedure

All cognitive tests which preceded the interventions were run at the beginning of the school year. They were conducted at the experimental lab of the Language and Cognition Unit at the Psychology Department of the University of Duisburg-Essen. Each participant completed the tests in the following order: Visual search task, WCST-64, recognition of prototypical faces and Stroop test, reversible images task, vigilance test. Interventions started immediately after the first cognitive tests had been completed and continued for a whole term; this resulted in 25 training sessions distributed over 18 weeks (interrupted by a 2-week holiday at the end of December and individual holidays). The mindfulness and concentration trainings took place twice a week, once for 60 min, once for 90 min, so that the children received roughly 150 min of treatment each week. Trainings were always held simultaneously as part of the regular class time. Every group was instructed by a team of at least two tutors. To avoid examiner effects, both the composition of the teams and the assignment of teams to intervention groups were changed repeatedly. The second set of cognitive tests was conducted immediately after interventions were finished.

## Results

Exploratory data analyses revealed differing base lines between the groups at pretest and differing error variances between and within groups (even though German secondary schools are supposed to host children of roughly homogeneous intellectual capacities, fifth graders as the entry groups are quite heterogeneous). Data were analyzed using linear mixed-effects modeling (except for the facial recognition task as explained below; Field et al., 2012). In order to check the effectiveness of the training interventions against the passive control group, each analysis included two planned contrasts with the passive control group as reference: First, no treatment (passive control group) vs. mindfulness training (experimental group), and second, no treatment (passive control group) vs. concentration training (active control group). Linear mixed-effect models were computed using R, Version 3.2.5 (R Core Team, 2016), and the function lme from the package nlme (Pinheiro, 2016). All models were built up from the following predictors: Participant, participant gender, time of measurement (pre- vs. post-test), group (mindfulness training vs. concentration training vs. passive control group), and the interaction of time with group. If further predictors were added, this is mentioned in the respective section. The following report of results focuses on those dependent variables that are central for testing our hypotheses. Due to the sample size and the pilot character of the present study the significance level was set at *p* ≤ 0.1. Significant contrasts are accompanied by effect sizes, which were computed as *r* = *t*^2^∕(*t*^2^ + *df*) (Field et al., 2012, p. 640).

### Sustained attention

Performance in the vigilance test was analyzed with respect to response accuracy and reaction time (RT) of hits. Reaction times shorter than 200 msec and longer than 2000 msec or more than three standard deviations (*SDs*) away from the mean were removed (1.4% of data). To determine response accuracy, yes- and no-responses were analyzed separately. For yes-responses, response accuracy was calculated by subtracting false alarms from hits. For no-responses, only misses were considered, since the task design did not include correct rejections. As the requirements on sustained attention increase over time and performance decreases with increasing length, a phenomenon known as vigilance decrement (MacLean et al., 2010), especially the second half of the vigilance test reflected the children's ability of sustained attention. Response accuracy for yes- and no-responses as well as RT were predicted from the above mentioned set of predictors (participant, participant gender, time of measurement (pre- vs. post-test), group (mindfulness training vs. concentration training vs. passive control group), and the interaction of time with group). Additional predictors in this model were test half and the interaction of test half with time and group.

Descriptive statistics on sustained attention are displayed in Table 1. As for **accuracy in yes-responses**, the linear mixed-effect model revealed a main effect of participant, $\chi_{\left(1\right)}^{2}$ = 6.30, *p* = 0.01, and a main effect of time of measurement, $\chi_{\left(1\right)}^{2}$ = 9.80, *p* = 0.002. Furthermore, there was a three-way interaction between test half, time of measurement and group, $\chi_{\left(3\right)}^{2}$ = 7.12, *p* = 0.07 (other *p*'s > 0.24). Respective contrasts indicated that the passive control group increased vigilance decrement from pre- to post-test, *b* = –4.59, *t*_(62)_ = –2.40, *p* = 0.02, *r* = 0.09, whereas for the mindfulness group vigilance decrement only tended to increase, *b* = −2.79, *t*_(62)_ = −1.54, *p* = 0.13, *r* = 0.11. This means that both groups deteriorated from pre- to post-test, but for the mindfulness training group this was only a tendency. The contrast regarding the concentration training group did not reach significance (*p* = 0.33). As for **accuracy in no-responses**, the analysis yielded a main effect of participant, $\chi_{\left(1\right)}^{2}$ = 10.32, *p* = 0.001, a main effect of test half, $\chi_{\left(1\right)}^{2}$ = 7.10, *p* = 0.008, and a three-way interaction between test half, time of measurement and group, $\chi_{\left(3\right)}^{2}$ = 8.81, *p* = 0.03 (other *p*'s > 0.44). Contrasts were significant for the passive control condition only, *b* = 1.24, *t*_(62)_ = 2.01, *p* = 0.05, *r* = 0.06 (other *p*'s > 0.54), indicating that the passive control group developed a more pronounced vigilance decrement from pre- to posttest, i.e., deteriorated performance. Regarding **RT**, the analysis revealed a main effect of test half, $\chi_{\left(1\right)}^{2}$ = 9.80, *p* = 0.002, in terms of a deceleration of RT from the first to the second test half for all groups (other *p*'s > 0.26).

**Table 1:** **Descriptive statistics on sustained attention, separated by measures (RA = response accuracy, RT = response time), treatment groups, and times of testing (T1 = pretest, T2 = posttest)**.

**Ability**	**Measure**	**Condition**	**T1**		**T2**	
			***M* (*SD*)**	**Median**	***M* (*SD*)**	**Median**
Vigilance test (entire test)	RA hits – false alarms	Mindfulness training	2.07 (11.14)	6.00	7.31 (8.37)	8.00
		Concentration training	–9.25 (19.90)	−4.00	0.88 (12.46)	4.00
		No intervention	4.70 (9.87)	9.50	7.00 (7.23)	7.50
	RA misses	Mindfulness training	9.81 (3.51)	9.50	8.44 (5.15)	8.00
		Concentration training	11.38 (3.66)	10.50	10.13 (4.73)	11.00
		No intervention	7.50 (3.54)	7.50	7.10 (2.56)	7.50
	RT hits	Mindfulness training	505.27 (38.18)	510.11	514.14 (39.43)	516.99
		Concentration training	516.07 (43.30)	488.75	510.80 (45.41)	513.87
		No intervention	503.83 (38.35)	506.35	507.21 (41.75)	495.16
Vigilance test (1st test half)	RA hits – false alarms	Mindfulness training	1.25 (7.59)	4.50	4.13 (5.61)	5.00
		Concentration training	–8.00 (14.88)	−3.00	1.63 (7.84)	3.00
		No intervention	3.40 (5.54)	4.00	5.80 (3.01)	7.00
	RA misses	Mindfulness training	4.50 (2.03)	4.00	4.00 (2.94)	3.50
		Concentration training	6.13 (2.53)	6.00	4.88 (2.90)	5.50
		No intervention	3.20 (1.75)	3.50	2.30 (1.83)	2.50
	RT hits	Mindfulness training	499.88 (45.44)	499.67	507.44 (57.55)	515.45
		Concentration training	511.48 (51.37)	522.50	509.76 (47.37)	516.80
		No intervention	488.02 (47.37)	493.08	493.21 (38.84)	481.25
Vigilance test (2nd test half)	RA hits – false alarms	Mindfulness training	0.81 (4.83)	1.00	3.18 (3.62)	2.50
		Concentration training	–1.25 (7.07)	1.50	–0.75 (6.86)	2.00
		No intervention	1.30 (4.83)	3.50	1.20 (4.71)	3.00
	RA misses	Mindfulness training	5.31 (2.06)	6.00	4.44 (2.56)	5.00
		Concentration training	5.25 (1.75)	5.00	5.25 (2.12)	5.00
		No intervention	4.30 (2.31)	4.00	4.80 (1.48)	4.50
	RT hits	Mindfulness training	520.05 (40.53)	524.06	516.95 (42.66)	511.81
		Concentration training	503.57 (84.63)	551.25	531.53 (35.00)	542.11
		No intervention	524.49 (57.57)	534.50	542.89 (53.55)	539.79

### Cognitive flexibility

Descriptive statistics on cognitive flexibility are displayed in Table 2. To analyze performance in the reversible figures task, the written solutions were assigned a score of zero when they did not involve any of the previously defined alternatives, a score of one when they involved one of the alternatives and of two when both alternatives were mentioned. Performance in this task was predicted from the above mentioned set of predictors, plus set of figures, and image. The analysis revealed a main effect of image, $\chi_{\left(1\right)}^{2}$ = 12.04, *p* < 0.001, but no other reliable effects (all other *p*'s > 0.20).

**Table 2:** **Descriptive statistics on cognitive flexibility, separated by measures, treatment groups, and times of testing (T1 = pretest, T2 = posttest)**.

**Measure**	**Condition**	**T1**		**T2**	
		***M* (*SD*)**	**Median**	***M* (*SD*)**	**Median**
Reversible figures	Mindfulness training	1.09 (0.35)	1.17	1.27 (0.31)	1.33
	Concentration training	1.17 (0.25)	1.08	1.35 (0.39)	1.42
	No intervention	1.05 (0.28)	1.00	1.22 (0.24)	1.17
Perseverative errors	Mindfulness training	9.00 (5.61)	6.00	6.75 (4.45)	6.00
	Concentration training	9.63 (5.48)	8.50	5.63 (3.66)	5.00
	No intervention	8.30 (3.23)	7.00	7.10 (3.00)	6.50

Sum score of perseverative errors in the WCST-64 (Kongs et al., 2000) served as another measure of cognitive flexibility. The analysis showed a main effect of time, $\chi_{\left(1\right)}^{2}$ = 9.15, *p* = 0.003, which suggested that all groups improved from pre- to posttest (other *p*'s > 0.35).

### Cognitive inhibition

Performance in the Stroop test was analyzed by contrasting incompatible with compatible trials. This accounts for individual differences in response behavior and focuses the difference between trials that involve conflict and require inhibition, and trials not involving conflict.

Descriptive statistics on cognitive inhibition are displayed in Table 3. To determine **response accuracy**, the number of correct incompatible trials was subtracted from the number of correct compatible trials. The linear mixed-effect model revealed a marginally significant main effect of gender, $\chi_{\left(1\right)}^{2}$ = 2.63, *p* = 0.11, indicating that, overall, girls tended to perform better than boys (other *p*'s > 0.33). **RT** values that differed more than three standard deviations from the mean were removed (1.72% of all correct trials). Only correct responses were included, and performance in compatible trials was subtracted from performance in incompatible trials. Analyses revealed a marginally significant interaction of time with group, $\chi_{\left(2\right)}^{2}$ = 4.44, *p* = 0.11 (other *p*'s > 0.27). Contrasts showed that the mindfulness training group improved whereas the passive control group deteriorated from pre- to posttest, *b* = −45.93, *t*_(31)_ = −1.69, *p* = 0.10, *r* = 0.08. The contrast comparing the concentration training group with the passive control group did not reach significance (*p* = 0.90).

**Table 3:** **Descriptive statistics on cognitive inhibition, separated by measures (RA = response accuracy, RT = response time), treatment groups, and times of testing (T1 = pretest, T2 = posttest)**.

**Measure**	**Condition**	**T1**		**T2**	
		***M* (*SD*)**	**Median**	***M* (*SD*)**	**Median**
Stroop RA correct compatible trials – correct incompatible trials	Mindfulness training	−9.19 (1.17)	−9.00	−9.56 (1.09)	−10.00
	Concentration training	−9.38 (0.74)	−9.50	−8.25 (4.40)	−10.00
	No intervention	−10.00 (0.67)	−10.00	−8.50 (4.20)	−9.50
Stroop RT correct incompatible – correct compatible trials	Mindfulness training	90.05 (56.99)	71.13	42.00 (71.92)	27.52
	Concentration training	94.33 (113.18)	92.27	88.24 (135.94)	78.09
	No intervention	77.64 (55.20)	83.62	125.42 (74.02)	131.25

### Data-driven information processing

Descriptive statistics on data-driven information processing are displayed in Table 4. Analysis of the facial recognition task focused on accuracy, as the number of correct responses was quite low, so that interpreting mean RT of correct responses would not have been sensible. Accuracy data were analyzed in a logistic regression model with gender, group, time of measurement, and stimulus (morphed from 2, 4, or 10 faces) as predictors, $\chi_{\left(11\right)}^{2}$ = 17.03, *p* = 0.15, *R*^2^ = 0.08 (Cox and Snell) 0.12 (Nagelkerke). The analysis revealed a main effect of time, *B* = −0.89, *SE* = 0.49, *p* = 0.07, as well as an interaction of group with time and stimulus, *Wald* = 8.00, *p* = 0.09 (other *p*'s > 0.14). Follow up contrasts showed a difference between the mindfulness and the concentration training group in responding to pictures morphed from 4 vs. 10 faces, *B* = 2.17, *SE* = 1.20, *p* = 0.07 (other contrasts: *p*'s > 0.38). Whereas, the concentration training group maintained performance from pre- to posttest for stimuli morphed from 4 to 10 faces, Fisher's exact test^7^: *p* = 1 in both cases, the mindfulness training group maintained performance for stimuli morphed from 4 faces, Fisher's exact test: *p* = 1, though improved from pre- to post-test for stimuli morphed from 10 faces, Fisher's exact test: *p* = 0.04.

**Table 4:** **Descriptive statistics on data-driven information processing, separated by measures (RT = response time), treatment groups, and times of testing (T1 = pretest, T2 = posttest)**.

**Measure**	**Condition**	**T1**			**T2**		
		**Frequency**			**Frequency**		
Face recognition false alarms 2-face morph	Mindfulness training	12			10		
	Concentration training	7			5		
	No intervention	5			4		
Face recognition correct rejections 2-face morph	Mindfulness training	4			6		
	Concentration training	1			3		
	No intervention	4			6		
Face recognition false alarms 4-face morph	Mindfulness training	12			12		
	Concentration training	3			7		
	No intervention	8			9		
Face recognition correct rejections 4-face morph	Mindfulness training	4			4		
	Concentration training	5			1		
	No intervention	1			1		
Face recognition false alarms 10-face morph	Mindfulness training	15			9		
	Concentration training	6			4		
	No intervention	7			7		
Face recognition correct rejections 10-face morph	Mindfulness training	1			7		
	Concentration training	2			4		
	No intervention	2			3		
Visual search false alarms blank trials	Mindfulness training	35			21		
	Concentration training	11			9		
	No intervention	29			18		
Visual search correct rejections blank trials	Mindfulness training	217			233		
	Concentration training	107			107		
	No intervention	124			137		
		***M*** **(*****SD*****)**		**Median**	***M*** **(SD)**		**Median**
Visual search RT blank trials	Mindfulness training	3990.44 (1227.49)		4059.74	3368.09 (924.38)		3308.07
	Concentration training	4368.20 (1056.65)		4734.95	4213.92(508.30)		4198.61
	No intervention	4060.75 (1232.39)		4118.45	3686.15(1228.51)		3564.25
		**Frequency**			**Frequency**		
Visual search misses unexpected position	Mindfulness training	19			20		
	Concentration training	11			8		
	No intervention	16			8		
Visual search hits unexpected position	Mindfulness training	107			108		
	Concentration training	53			55		
	No intervention	64			72		
		***M*** **(*****SD*****)**	**Median**		***M*** **(*****SD*****)**	**Median**	
Visual search RT unexpected position	Mindfulness training	51.41 (749.83)	23.69		−251.05 (329.27)	−275.35	
	Concentration training	398.17 (479.20)	386.20		146.00 (277.42)	120.77	
	No intervention	679.45 (1065.61)	450.62		−33.08 (709.31)	−246.69	

Performance in the visual search task as second measure of data-driven information processing was analyzed separately for blank trials and pictures containing the target in an unexpected position, as these were the conditions requiring data-driven information processing. Regarding RTs, 2.07% of the data were removed due to pressing of a wrong key and 5.1% due to differences of more than two standard deviations from the mean. In the case of RT the standard set of predictors was complemented by image and set of images.

Regarding **response accuracy in blank trials**, i.e., images that did not comprise the target and therefore required a no-response, analyses of **correct rejections** showed a marginally significant main effect of time, $\chi_{\left(1\right)}^{2}$ = 2.51, *p* = 0.11, suggesting that participants of all groups improved performance from pre- to posttest (other *p*'s > 0.24). As for **false alarms**, there was a tendency for a main effect of time, $\chi_{\left(1\right)}^{2}$ = 2.24, *p* = 0.13, which indicated that, on average, performance tended to improve from pre- to posttest in all groups (other *p*'s > 0.19). Regarding **RT** in blank trials, the linear mixed-effect model revealed a significant main effect of time, $\chi_{\left(1\right)}^{2}$ = 18.10, *p* < 0.001, and an interaction of time with group, $\chi_{\left(2\right)}^{2}$ = 6.85, *p* = 0.03 (other *p*'s > 0.38). Respective contrasts revealed that the passive control group improved to a larger degree than the concentration training group, *b* = 283.83, *t*_(158)_ = 2.14, *p* = 0.03, *r* = 0.02, and that the mindfulness training group improved to a larger degree than the passive control group, *b* = –257.27, *t*_(158)_ = –2.34, *p* = 0.02, *r* = 0.03.

For **pictures containing the target in an unexpected position**, response accuracy for hits and misses was analyzed separately. In terms of **hits**, analyses showed a marginally significant main effect of gender, $\chi_{\left(1\right)}^{2}$ = 2.53, *p* = 0.11, with girls tending to perform better than boys (other *p*'s > 0.22). As for **misses**, the analysis again revealed a tendency toward a main effect of gender, $\chi_{\left(1\right)}^{2}$ = 2.34, *p* = 0.13, indicating a superior performance of girls over boys (other *p*'s > 0.22). **RT**s were corrected for possible influences of target size and distance to the fixation point upon stimulus onset by means of a regression analysis, with RT as dependent variable and size of target as well as distance to the center as predictors. The resulting unstandardized residual served as cleaned RT. The linear mixed-effect model revealed a significant main effect of group, $\chi_{\left(1\right)}^{2}=$ 5.25, *p* = 0.07. Both contrasts comparing each treatment group with the passive control group were insignificant, all *p's* > 0.25. Furthermore, there was a significant main effect of time, $\chi_{\left(1\right)}^{2}$ = 13.33, p < 0.001, indicating improvements for all groups between times of measurement (other *p*'s > 0.57).

## Discussion

The aim of the present pilot study was to analyze cognitive effects of mindfulness training with a design that referred to a sound theoretical framework and included an active and a passive control group as well as a broad range of objective measures. Our first hypothesis postulated that both mindfulness and concentration training are associated with higher improvements regarding sustained attention, cognitive flexibility, cognitive inhibition, and data-driven information processing than schooling and maturation only. The second hypothesis assumed that mindfulness training yields specific cognitive benefits, compared to a different type of cognitive training. The first hypothesis was partly confirmed in accuracy measures in the vigilance test where the passive control group performed worse than both training groups. In all other measures that showed differences between groups, i.e., Stroop task: RT, recognition task: Correct rejections of morphed faces, and in the visual search task: RT in blank trials, the concentration training group showed equal, or worse performance when compared to the passive control group. This suggests that the concentration training did not consistently benefit the cognitive abilities under investigation beyond maturation and schooling. The rather moderate effects of the concentration training may be related with the period of school career at which the sample was investigated. German fifth graders have just left their primary schools and face a totally new educational environment in their first grade of secondary school, which at Gymnasium is at the same time more furthering and more challenging than at primary school. This may lead to a fast increase of cognitive abilities in the fifth grade leaving little room for further improvement through additional concentration training. Regarding the second hypothesis, the reported pattern of results therefore supports the idea of an advantage for the mindfulness group compared to the concentration training group in improved cognitive abilities.

However, some tests did not show any group differences (reversible figures task, WCST). We will therefore explore in more detail to what extent the mindfulness training led to specific improvements of particular cognitive abilities as predicted by Bishop et al. (2004). The evidence at hand suggests distinctive benefits of mindfulness training for **cognitive inhibition** and **data-driven information processing**: If the respective measures showed advantages for one of the groups, they were specifically and beneficially affected by mindfulness training (i.e., RT in the Stroop task, correct rejection of prototypical faces, and RT in blank trials in the visual search paradigm). These findings are in line with earlier findings obtained with adult samples (Chan and Woollacott, 2007; Moore and Malinowski, 2009; Zanesco et al., 2013) and suggest that mindfulness trainings can enhance these cognitive abilities not only for adults but also for fifth graders. Several measures of these cognitive abilities which did not show systematic group differences, revealed an improvement for all groups over time (visual search: Accuracy in blank trials, response times for objects in unexpected positions) indicating that more difficult tasks or more difficult stimuli might be employed in future studies to even better differentiate between groups. Regarding **sustained attention**, our results are less clear. Regarding RT, none of the groups changed performance from pre- to posttest. As for accuracy, the passive control group deteriorated from pre- to post-test, whereas the concentration training group maintained performance. Performance of the mindfulness training group resembled the concentration training group except from a marginal tendency to deteriorate accuracy of yes-responses. Previous studies generally found sustained attention to profit by intensive mindfulness retreats but not by less intensive MBSR courses (cf. Overview of existing studies). The lack of a clear mindfulness-based improvement in the present study could be due to the fact that the training was not intense enough to consistently affect this cognitive ability. However, it should be kept in mind that on the whole, sustained attention performance of the mindfulness training group was still better than that of the passive control group. The two measures of **cognitive flexibility** did not reflect a specific effect of mindfulness practice: Performance both in the reversible figures task and the Wisconsin Card Sorting Task developed comparably in all groups. This may reflect an effect of schooling and an increase of academic achievements in the fifth grade compared to primary school (see above). Again, more difficult tasks should be employed in future studies to avoid ceiling effects and to detect potential specific benefits of mindfulness practice for this cognitive skill.

Finally, for several measures our analyses revealed a tendency across groups for girls to perform better than boys (Stroop: Accuracy, Visual search task: Accuracy in detecting objects in unexpected positions). From previous research no hypotheses about gender differences could be derived and they were not in the focus of our study. Moreover, the tendency in the present results does not indicate a differential benefit for girls through training. However, this issue might be followed up in future research with a larger sample and equal gender distributions in experimental conditions.

Albeit preliminary, the results of the present investigation suggest specific effects of mindfulness training with fifth graders on cognitive inhibition and data-driven information processing. Nevertheless, the findings should be treated with care, because they are grounded on a small and quite heterogeneous sample. Furthermore, the evidence is limited by the fact that the passive control group was not randomized. However, the pattern of results encourages further studies with larger randomized and controlled trials to test the replicability of effects. After successful replication, the mechanisms underlying these cognitive effects deserve further empirical investigation.

From a neurodevelopmental perspective, the suggested improvements in cognitive inhibition are of special interest, as they have potential implications for self-regulation. Adolescents tend to display poorer inhibition and more impulsive, risk taking behaviors. Self-regulatory abilities may be particularly affected by maturational changes during development. Emotions continuously compete with cognitive processes for attention, and prefrontal attentional control systems are responsible for the inhibition and monitoring of emotions in need of regulation (Ochsner and Gross, 2005). This means that improvements in cognitive inhibition, which according to these findings can be achieved through mindfulness training, could strengthen the self-regulatory abilities of adolescents. Future studies with adolescents should therefore include measures and paradigms that explicitly tap emotion regulation.

A validation of our results with students from different schools and other age groups would also be desirable to strengthen the evidence. It should be noted, though, that the present study was conducted with children from diverse national, cultural, and religious backgrounds. In case of successful replication and in view of the existing evidence concerning positive cognitive effects of mindfulness interventions, there would be good reasons to establish mindfulness interventions in the school context.

## Author contributions

The authors contributed to the work in the following ways: Substantial contributions to the conception of the work: LS, LW, SB; acquisition, analysis, or interpretation of data for the work: LW, LS; Drafting the work or revising it critically for important intellectual content: LS, LW, SB; Final approval of the version to be published: LW, LS; SB; Agreement to be accountable for all aspects of the work in ensuring that questions related to the accuracy or integrity of any part of the work are appropriately investigated and resolved: LW, LS, SB.

## Funding

The research reported here was supported by a Mercator Research Center Ruhr grant to LS and LB, grant number An-2013-0028. The funding source was neither involved in the study design, nor in the collection, analysis, and interpretation of data, in the writing of this report or in the decision to submit the article for publication.

### Conflict of interest statement

The authors declare that the research was conducted in the absence of any commercial or financial relationships that could be construed as a potential conflict of interest.

## Footnotes

^1^After 4 years of elementary school, children in Germany attend different kinds of secondary schools depending on their academic performance. Gymnasium is the type of school that prepares students for university entrance. The fifth graders in our sample are students in their first year of Gymnasium (hence fifth year of schooling) and are aged about 10–11 years.

^2^In this test, participants count objects from two semantic categories that are presented one by one in random order (Chambers et al., 2008).

^3^These interventions comprised a training similar to MBSR plus mindfulness practices for military personnel in the stage of predeployment (Jha et al., 2010) or focused attention meditations and group discussions in eight 45-min sessions and daily 10-min meditations out of class (Mrazek et al., 2013).

^4^This program includes breathing exercises, physical, and sensory activities as well as thought and communication exercises. It was practiced in 45-min sessions that took place bimonthly over a period of 24 weeks.

^5^The Wisconsin Card Sorting Test measures a variety of executive functions. However, the perseverative component, which is represented by so-called perseverative errors, reflects “the ability to shift response set and respond flexibly to changing test requirements” (Kongs et al., 2000, p. 44).

^6^There are also reviews that report no effects on depression and anxiety in various clinical populations (Toneatto and Nguyen, 2007), or only small effects on these variables in a population with chronic somatic diseases (Bohlmeijer et al., 2010), respectively.

^7^Fisher's exact test was used due to expected cell frequencies of <5.
